# Supplementary material for: INKA, an integrative data analysis pipeline for phosphoproteomic inference of active kinases
Source: Mol Syst Biol. 2019 Apr 12;15(4):e8250. doi: 10.15252/msb.20188250 (PMC6461034; doi:10.15252/msb.20188250)
Supplement: Supplementary file 1 — Appendix [file MSB-15-e8250-s001.pdf]

APPENDIX

MSB-18-8250RRR, *INKA, an integrative data analysis pipeline for phosphoproteomic inference of active kinases*

Table of Contents

Item	Title	Page
Appendix Figure S1	Coverage of 538 unique protein kinases by resources providing kinase-substrate relationships.	2
Appendix Figure S2	Kinase-substrate relation network for top 20 INKA-scoring kinases and their observed substrates in K-562 chronic myelogenous leukemia cells with a BCR-ABL fusion.	3
Appendix Figure S3	Kinase-substrate relation network for top 20 INKA-scoring kinases and their observed substrates in SK-Mel-28 melanoma cells with BRAFV600E.	4
Appendix Figure S4	Kinase-substrate relation network for top 20 INKA-scoring kinases and their observed substrates in HCC827-ER3 non-small cell lung carcinoma cells.	5
Appendix Figure S5	Kinase-substrate relation network for top 20 INKA-scoring kinases and their observed substrates in H2228 non-small cell lung carcinoma cells with an EML4-ALK fusion.	6
Appendix Figure S6	Correlation between kinase-specific INKA scores and estimated p-values.	7
Appendix Figure S7	Comparison of INKA analysis versus KARP analysis of phosphoproteomics data on oncogene-driven cell lines, and on wild-type and EGFR-mutant U87 cells ± erlotinib treatment.	8-9
Appendix Figure S8	INKA plot and top 20 INKA-scoring kinase bar graph for HCC827 cells with mutant EGFR.	10
Appendix Figure S9	INKA components in differential analysis.	11
Appendix Figure S10	MS intensity-based INKA analysis of data published by Bensimon et al. on TiOx-captured phosphoproteomes from G361 melanoma cells following radiomimetic treatment.	12
Appendix Figure S11	INKA analysis of patient-derived xenograft (PDX) tissue subjected to pTyr-based and TiOx-based phosphoproteomics.	13-17
Appendix Figure S12	Comparison of count-based versus intensity-based INKA analysis of phosphoproteomics data on oncogene-driven cell lines.	18
Appendix Figure S13	iBAQ-based INKA analysis of phosphoproteomics data on oncogene-driven cell lines.	19-21
Appendix Figure S14	INKA analysis of 11-plex TMT phosphoproteomics data of ALK signalling in neuroblastoma cells.	22-24

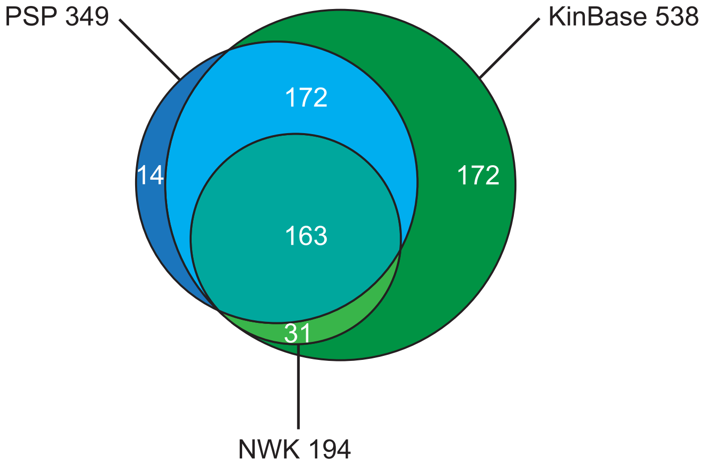

Appendix Figure S1. Coverage of 538 unique protein kinases by resources providing kinase-substrate relationships.

The Venn diagram shows that 172 kinases in KinBase are missing from both PhosphoSitePlus (PSP) and NetworKIN (NWK), 31 are only covered by NWK, 172 are only covered by PSP, while 163 are covered by both NWK and PSP. Additionally, 14 proteins annotated by PSP are not present in KinBase, but mostly involve small-molecule kinases or proteins with spurious (electronic) annotation, with the exception of two fusion protein species with established protein kinase activity (BCR-ABL and NPM-ALK).

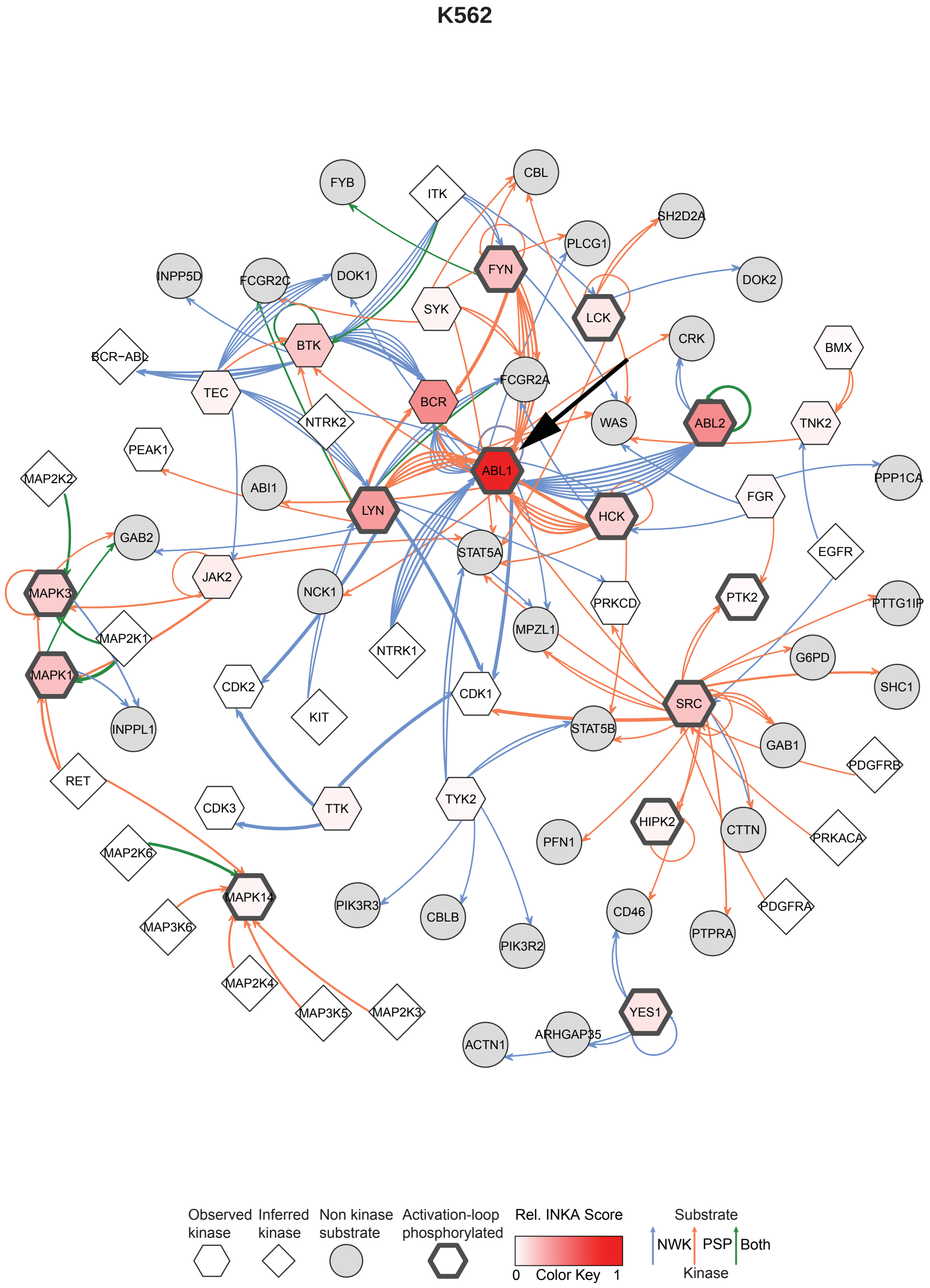

Appendix Figure S2. Kinase-substrate relation network for top 20 INKA-scoring kinases and their observed substrates in K-562 chronic myelogenous leukemia cells with a *BCR-ABL* fusion.

ABL1 is a highly connected and central node. Kinases downstream of BCR-ABL signaling, such as SRC, are also active, albeit to a lower extent.

Data information: Data are plotted for single-sample measurement.

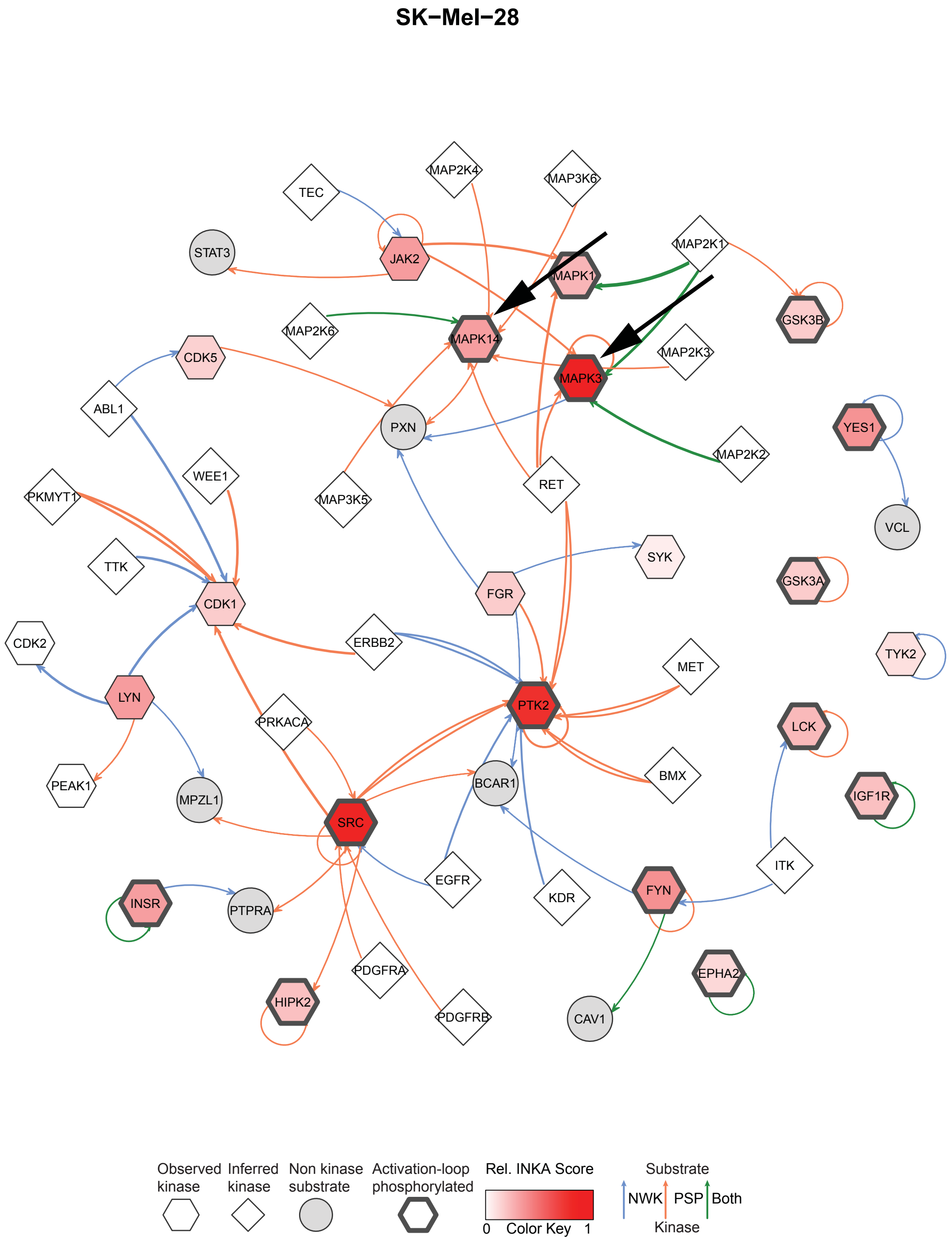

Appendix Figure S3. Kinase-substrate relation network for top 20 INKA-scoring kinases and their observed substrates in SK-Mel-28 melanoma cells with BRAF^{V600E}.

Two clusters of activated kinases are observed, one containing BRAF targets MAPK1 and MAPK3, and the other containing SRC as highly connected nodes.

Data information: Data are plotted for single-sample measurement.

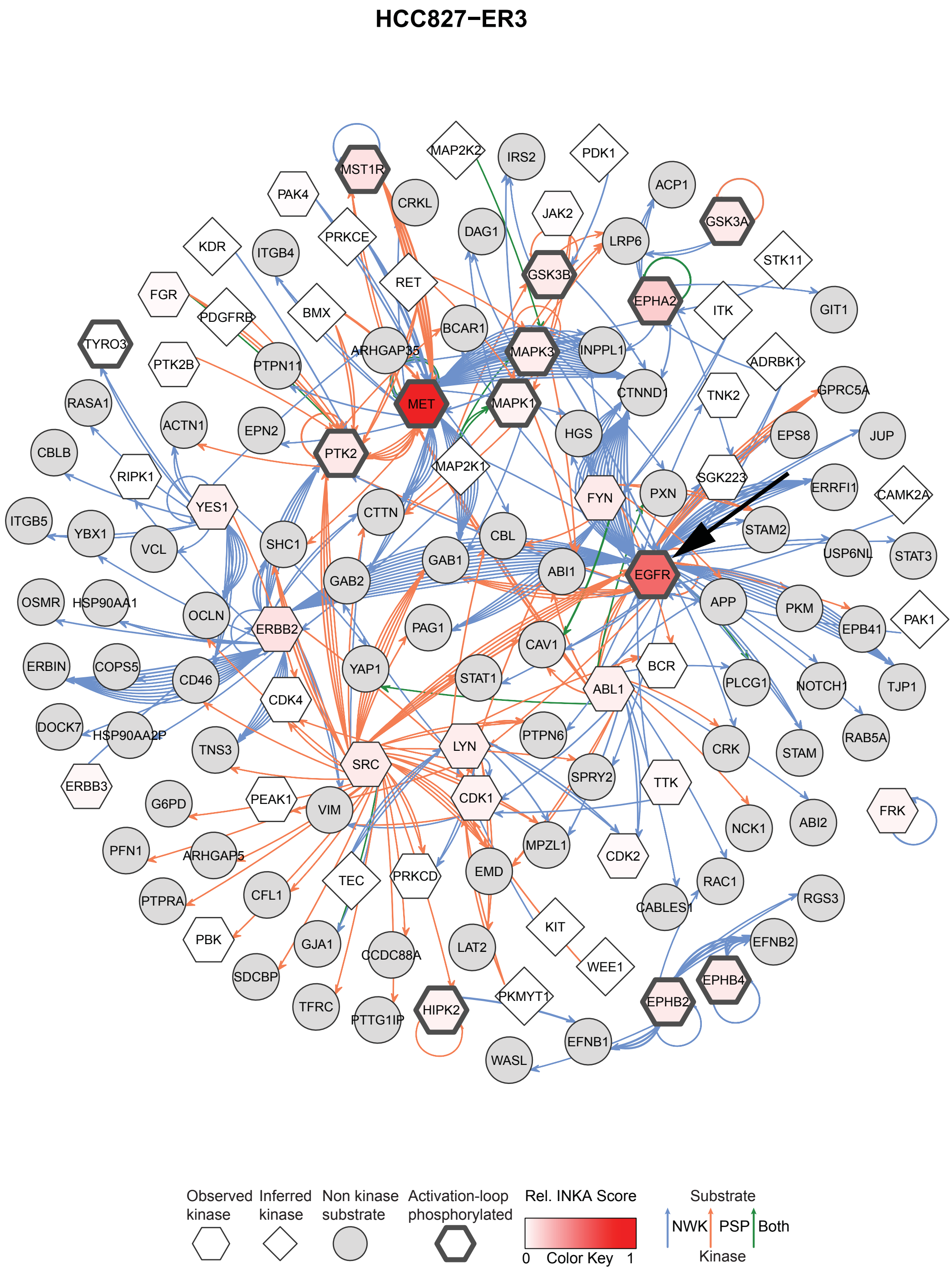

Appendix Figure S4. Kinase-substrate relation network for top 20 INKA-scoring kinases and their observed substrates in HCC827-ER3 non-small cell lung carcinoma cells.

EGFR and MET are central and highly connected nodes. AXL, associated with erlotinib resistance of HCC827-ER3 cells, is missed by INKA score-based analysis as no substrate-centric data is available for this kinase.

Data information: Data are plotted for single-sample measurement.

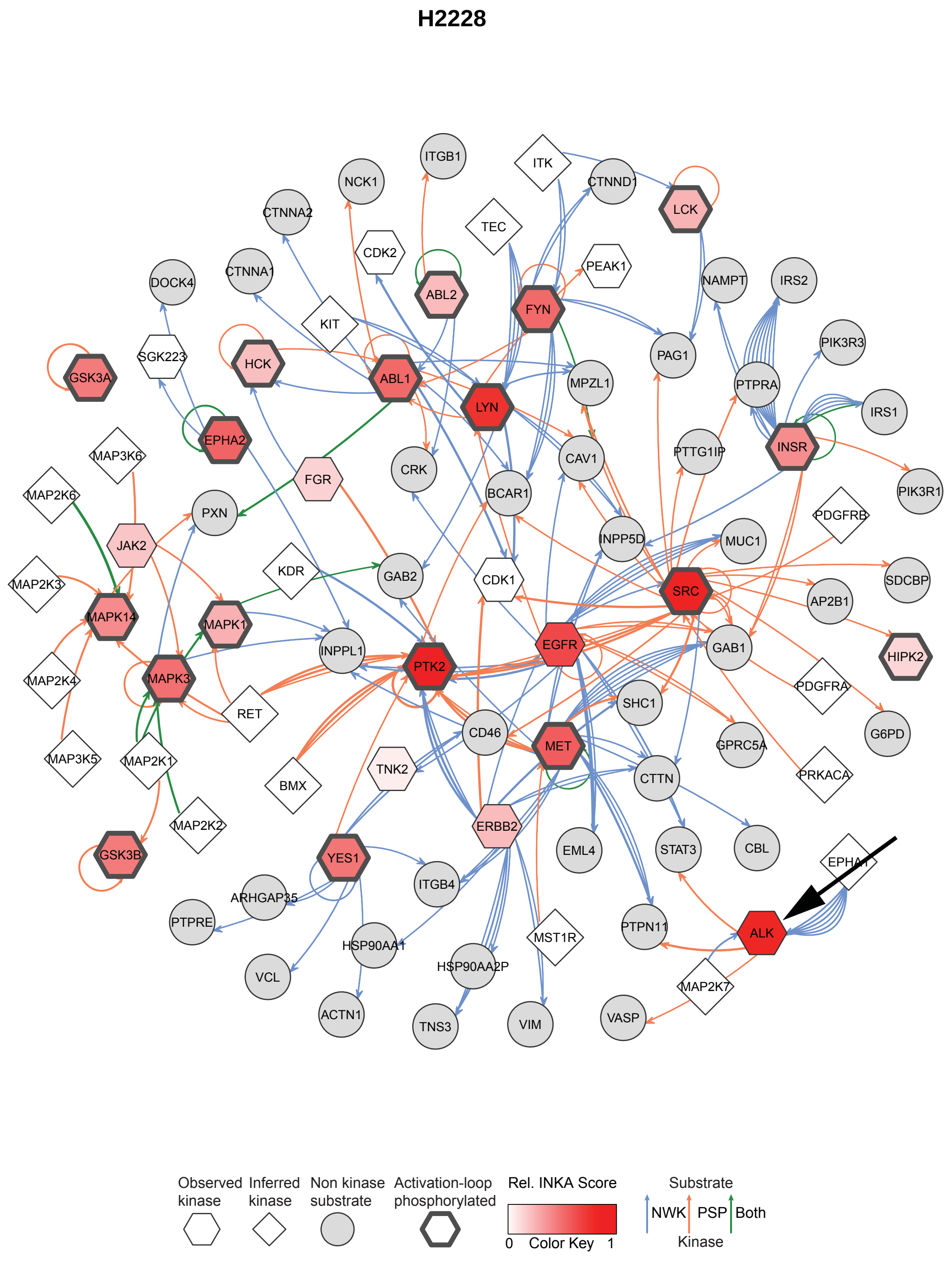

Appendix Figure S5. Kinase-substrate relation network for top 20 INKA-scoring kinases and their observed substrates in H2228 non-small cell lung carcinoma cells with an *EML4-ALK* fusion.

Multiple highly active and connected nodes are present in the network for H2228, implying relative insensitivity to inhibition of ALK alone, in line with previous functional data. Dual inhibition of the number-5 hyperactive node, EGFR, and ALK results in significant reduction of proliferation(Voena *et al*, 2013).

Data information: Data are plotted for single-sample measurement.

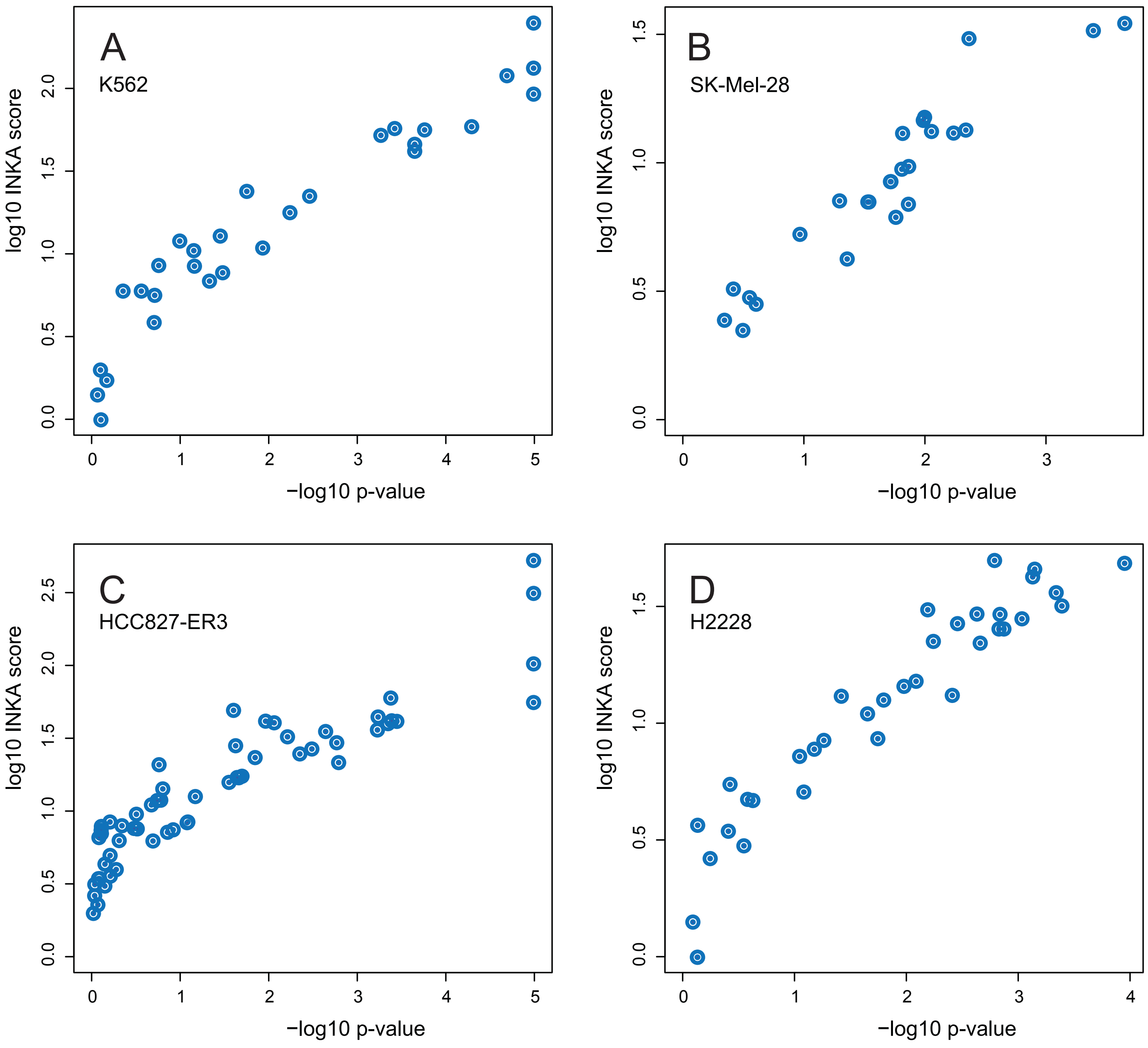

Appendix Figure S6. Correlation between kinase-specific INKA scores and estimated p-values.

Log10-transformed INKA scores are plotted against (sign-switched) log10-transformed p-values for each of four cell line use cases. Highly significant, positive Spearman rank correlation coefficients for each of the cell lines demonstrate a monotonic trend: higher INKA scores are associated with lower p-values.

A K562 chronic myelogenous leukemia cells.
B SK-Mel-28 melanoma cells.
C HCC827-ER3 lung cancer cells.
D H2228 lung cancer cells.

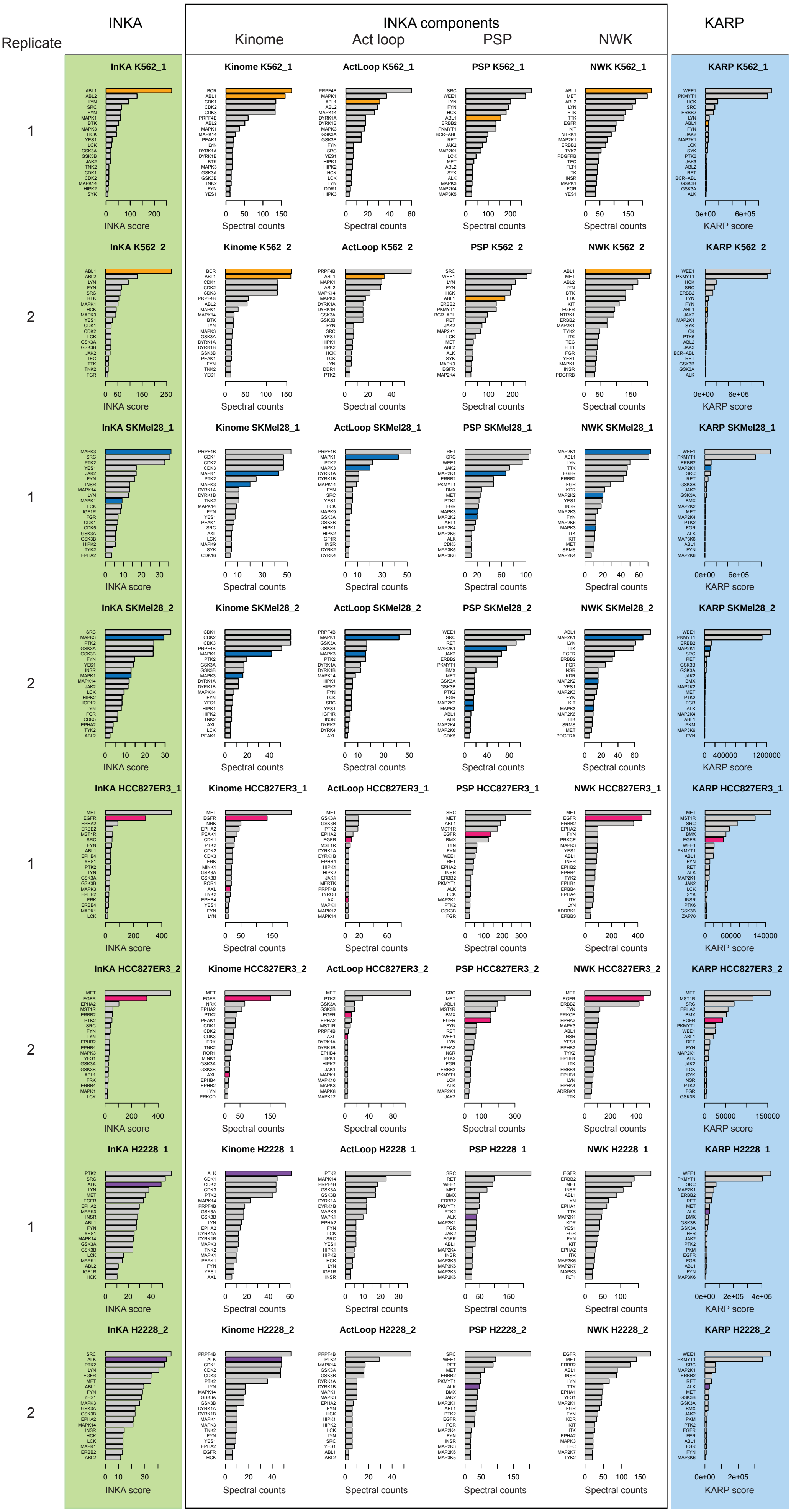

Appendix Figure S7A. Comparison of INKA analysis versus KARP analysis of phosphoproteomics data on oncogene-driven cell lines.

Phosphotyrosine IP data for K562, SK-Mel-28, HCC827-ER3 and H2228 were analysed by INKA (green column) and KARP (blue column). Also included are the results for the individual components of the INKA analysis (Kinome, Activation Loop, PSP, and NWK; white columns).

Data information: there are duplicates for each cell line.

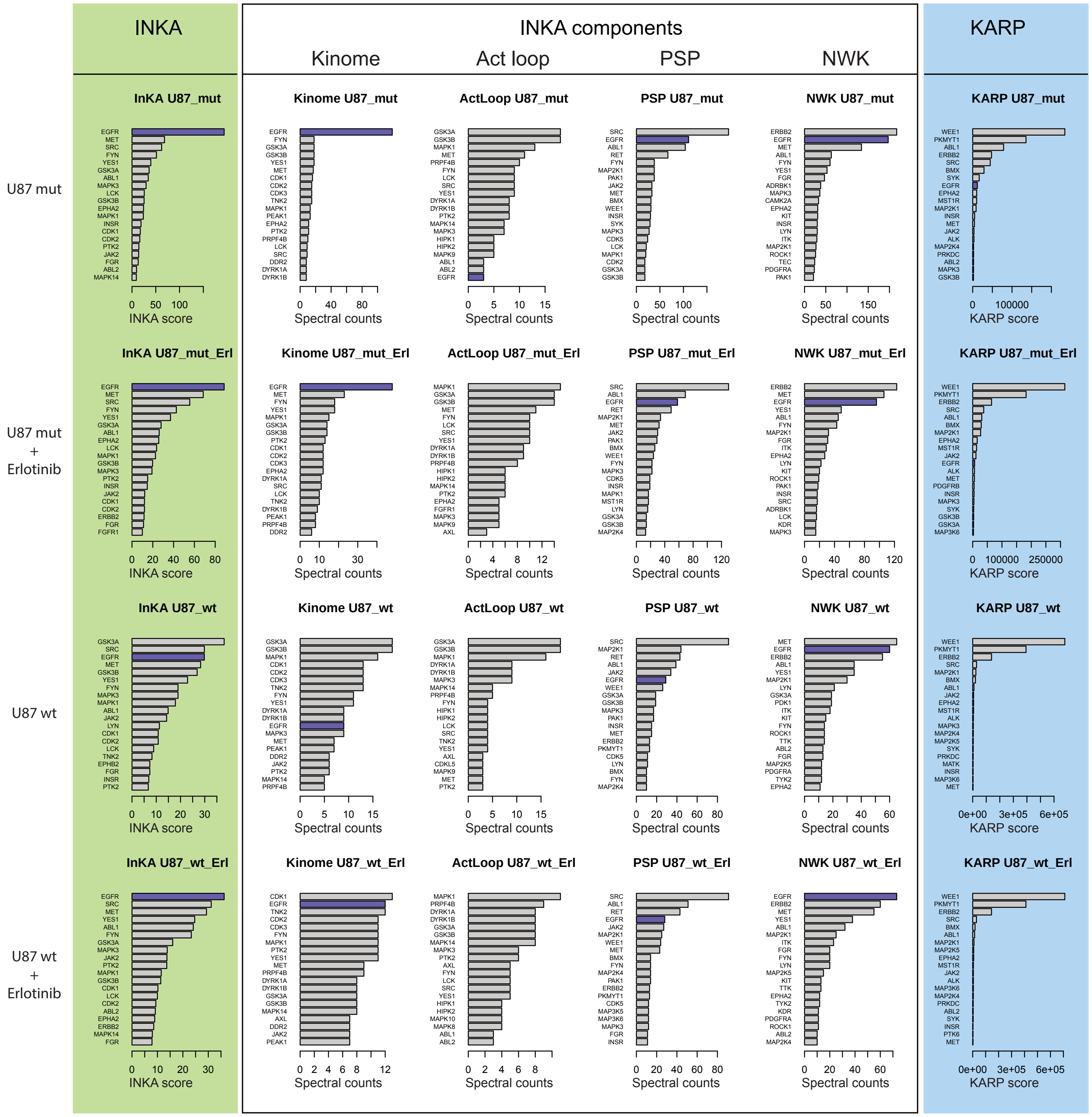

Appendix Figure S7B. Comparison of INKA analysis versus KARP analysis of phosphoproteomics data on wild-type and EGFR-mutant U87 cell lines with or without erlotinib treatment.

Phosphotyrosine IP data for wild-type U87 (wt) cells and U87-EGFRvIII (mut) cells with or without erlotinib treatment for 2h were analysed by INKA (green column) and KARP (blue column). Also included are the results for the individual components of the INKA analysis (Kinome, Activation Loop, PSP, and NWK; white columns).

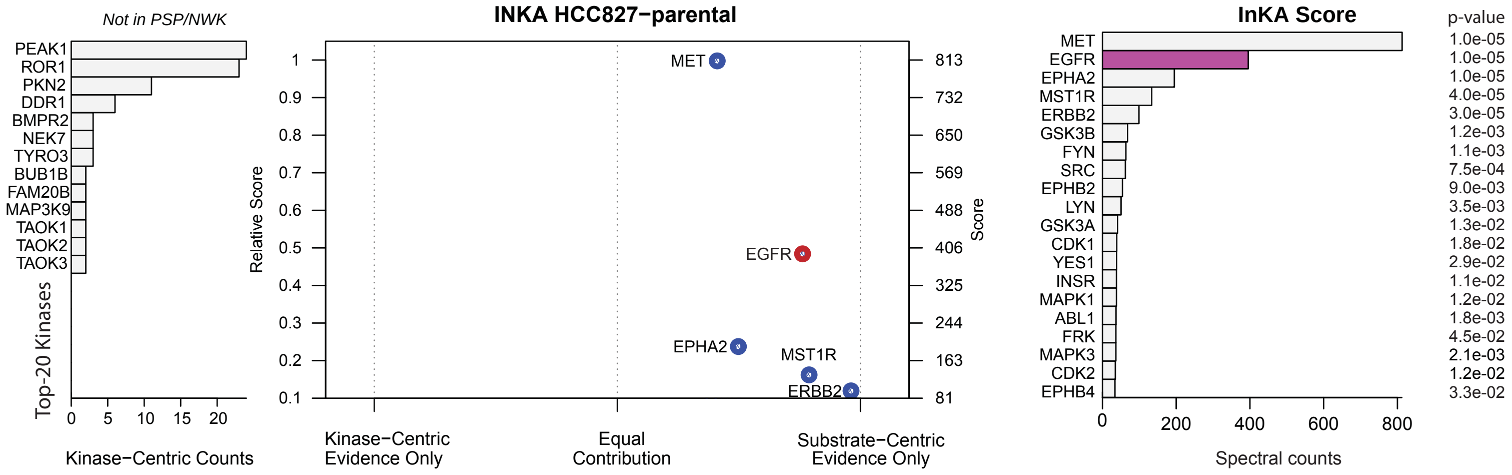

Appendix Figure S8. INKA plot and top 20 INKA-scoring kinase bar graph for HCC827 cells with mutant *EGFR*.

In the INKA plot proper, the vertical position of kinases (driver in red) is determined by their INKA score, whereas the horizontal position is determined by the (im)balance of evidence from kinase-centric and substrate-inferred arms of the analysis. EGFR and MET are implicated as the most active kinases. Kinases not covered by PhosphoSitePlus (PSP) and NetworKIN (NWK) are visualized in a flanking bar graph on the left. Note absence from the bar graph of the AXL kinase that is responsible for erlotinib resistance in the HCC827-ER3 sub-line (see Fig **3**c).

Data information: Data are plotted for single-sample measurement.

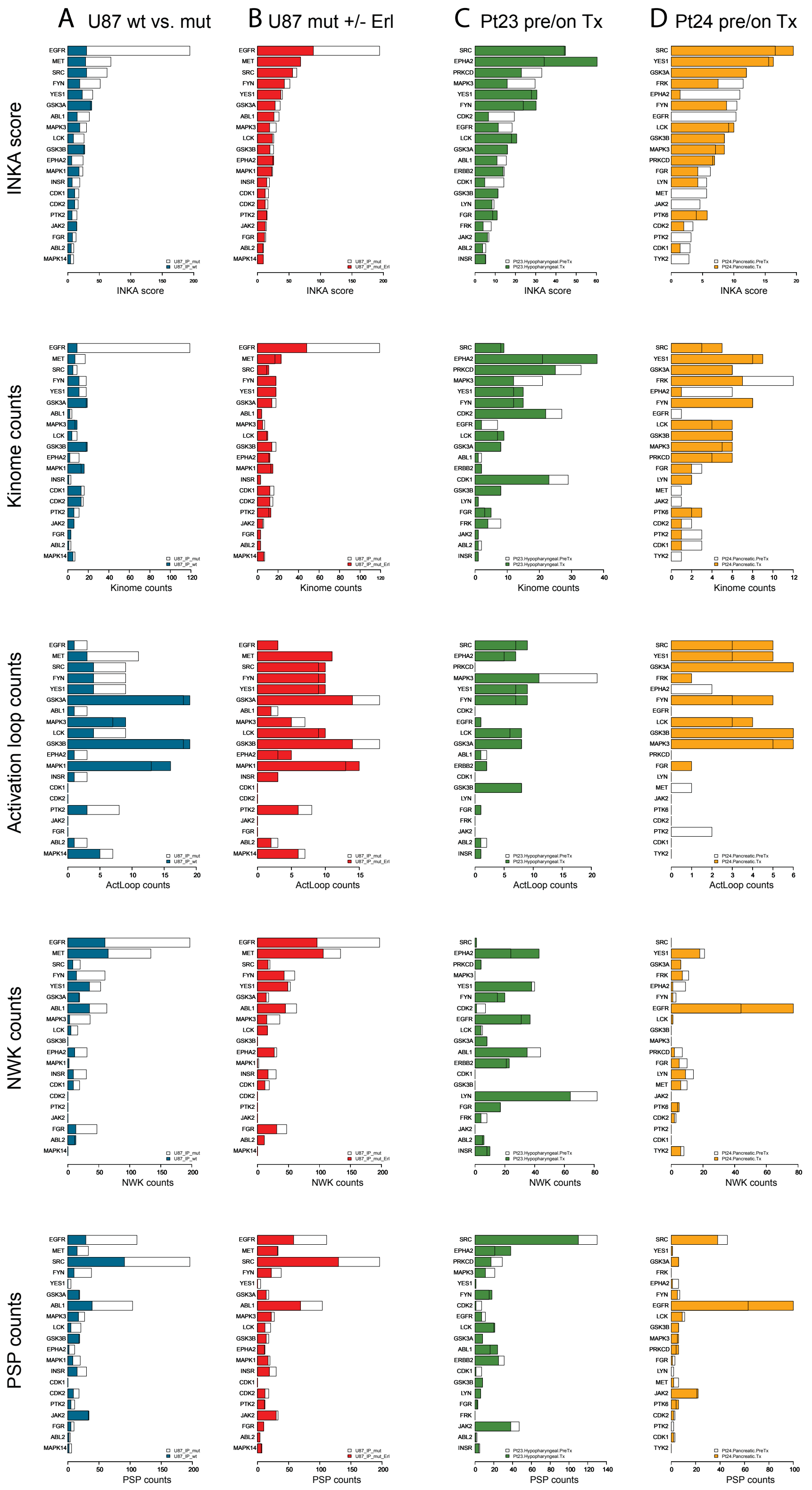

Appendix Figure S9. INKA components in differential analysis.

A U87 EGFRvIII mutant glioblastoma cells (white) versus U87 wild-type cells (blue).

B U87 EGFRvIII mutant glioblastoma cells that were not treated (white) versus treated with erlotinib (red).

C Biopsy of Patient 23 pre- and on erlotinib treatment

D Biopsy Patient 24 pre- and on erlotinib treatment

Abbreviations: Erl, erlotininb.

Data information: Data are plotted for single-sample measurements.

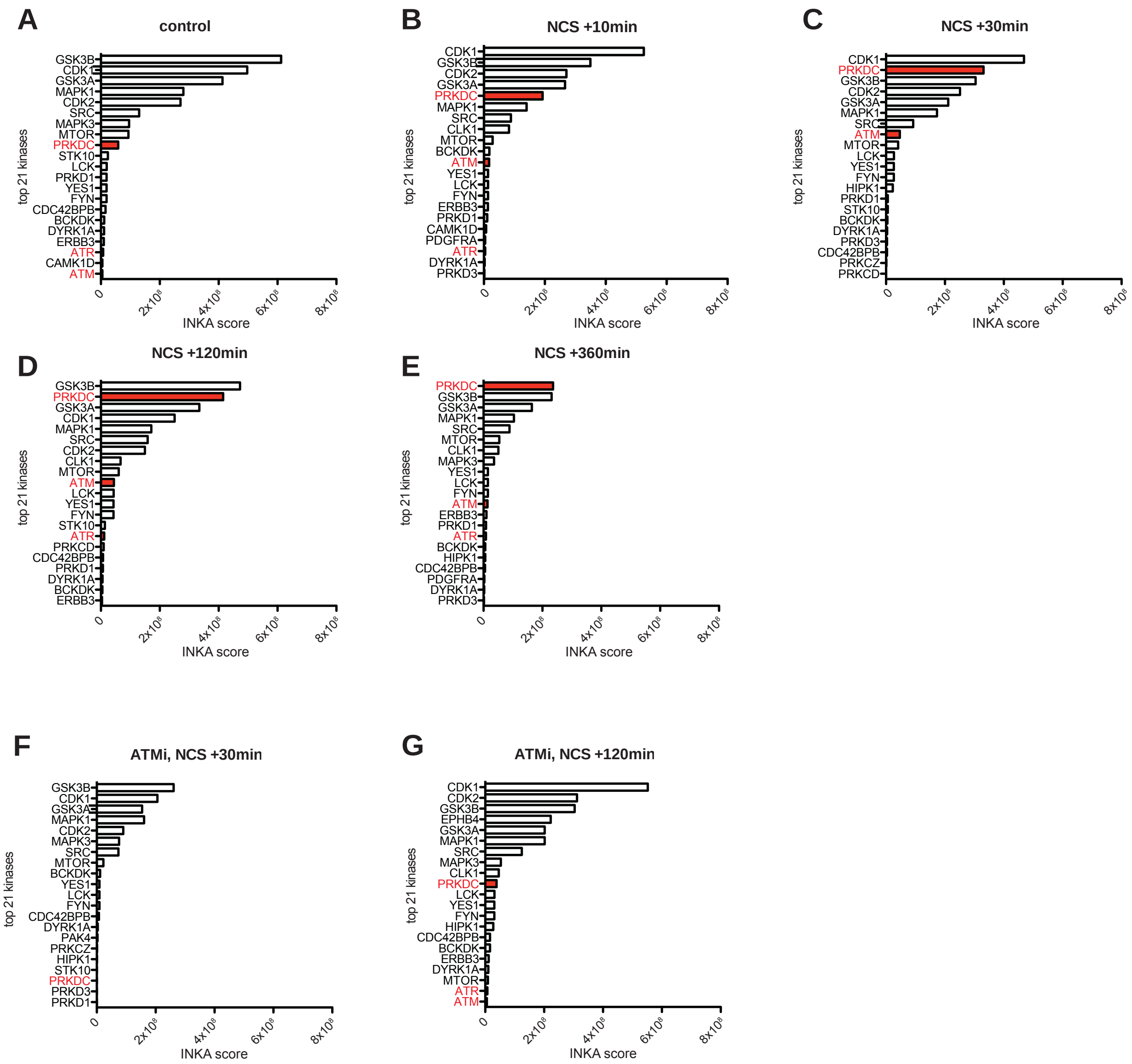

Appendix Figure S10. MS intensity-based INKA analysis of published data on TiOx-captured phosphoproteomes from G361 melanoma cells following radiomimetic treatment (Bensimon *et al.*).

A INKA score bar graphs for G361 at baseline.

B-E INKA score bar graphs for G361 after 10 min (B), 30 min (C), 120 min (D), or 360 min (E) treatment with 200 ng/ml neocarzinostatin (NCS).

F,G INKA score bar graphs for G361 after 30 min (F) or 120 min (G) treatment with 200 ng/ml neocarzinostatin in the presence of 10 μM KU55933 (ATM inhibitor, ATMi).

DNA damage-induced kinases ATM, ATR, and PRKDC/DNA-PK are highlighted in red.

Data information: Data are plotted for single-sample measurement.

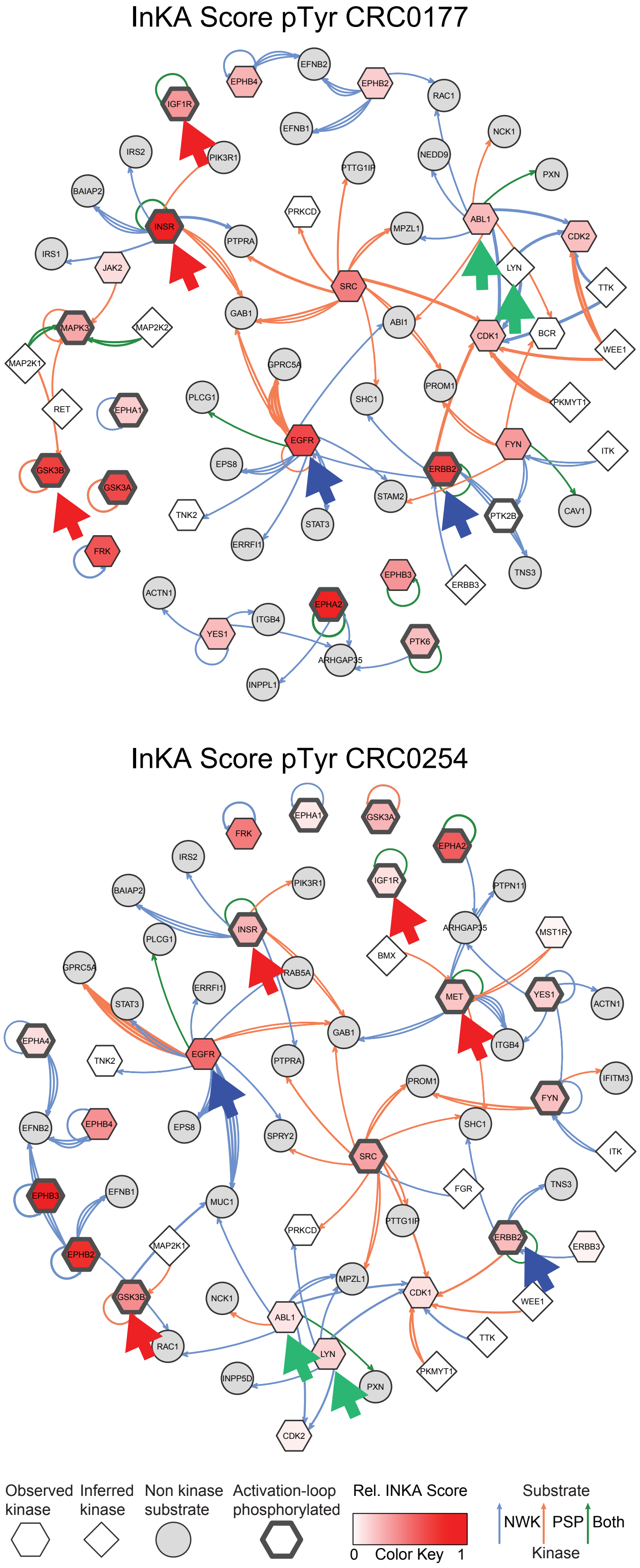

Appendix Figure S11A. INKA analysis of patient-derived xenograft (PDX) tissue subjected to pTyr-based and TiOx-based phosphoproteomics.

Kinase-substrate relation networks generated using pTyr-based phosphoproteomic data for PDX models CRC0177 (upper) and CRC0254 (lower) for metastatic colorectal cancer. Large arrows indicate targets for afatinib (blue), BMS-754807 (red), and imatinib (green), respectively.

Data information: Data are plotted for single-sample measurement.

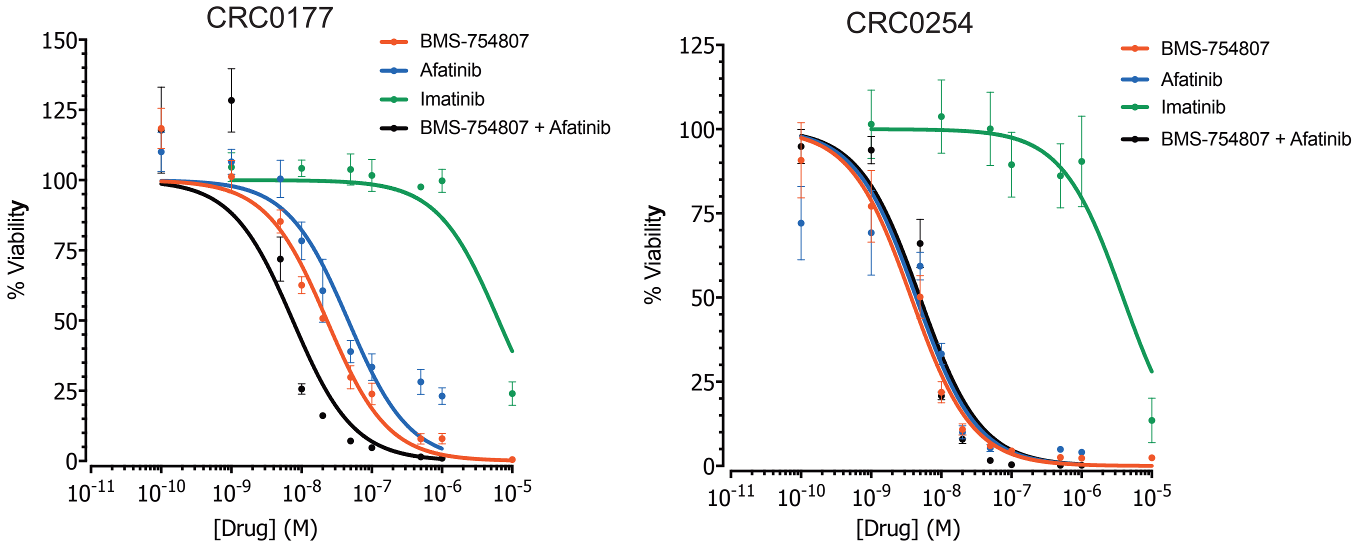

	IC50	
**BMS-754807**	25 nM	CI95: (18.2 nM to 29.5 nM)
**Afatinib**	50 nM	CI95: (33.9 nM to 64.6 nM)
**BMS-754807 + Afatinib**	8 nM	CI95: (5.1 nM to 11.0 nM)
**Imatinib**	6 µM	CI95: (5.0 µM to 8.7 µM)

	IC50	
**BMS-754807**	4 nM	CI95: (2.8 nM to 5.0 nM)
**Afatinib**	4 nM	CI95: (2.8 nM to 6.6 nM)
**BMS-754807 + Afatinib**	5 nM	CI95: (3.7 nM to 6.5 nM)
**Imatinib**	4 µM	CI95: (2.7 µM to 5.8 µM)

Appendix Figure S11B. INKA analysis of patient-derived xenograft (PDX) tissue subjected to pTyr-based and TiOx-based phosphoproteomics.

Dose-response curves for drug-treated organoids derived from PDX tissue. Organoids were derived from models CRC0177 and CRC0254, cultured for 2-3 weeks, treated with a single drug (blue: afatinib, red: BMS-754807, green: imatinib) or the combination BMS-754807 + afatinib (black) for 7 days and metabolic activity was measured by a luminiscent ATP assay as a correlate of cell viability.

Data information: Data are for replicates from pooled experiments after data normalization: 47 replicates/5 experiments (control), 16 replicates/3 experiments (BMS-54807), 13 replicates/3 experiments (afatinib), 6 replicates/1 experiment (combination) and 6 replicates/2 experiments (imatinib) for CRC0177, and 17 replicates/4 experiments (control), 12 replicates/2 experiments (BMS-54807), 3 replicates/1 experiment (afatinib), 3 replicates/1 experiment (combination) and 6 replicates/2 experiments (imatinib) for CRC0254.

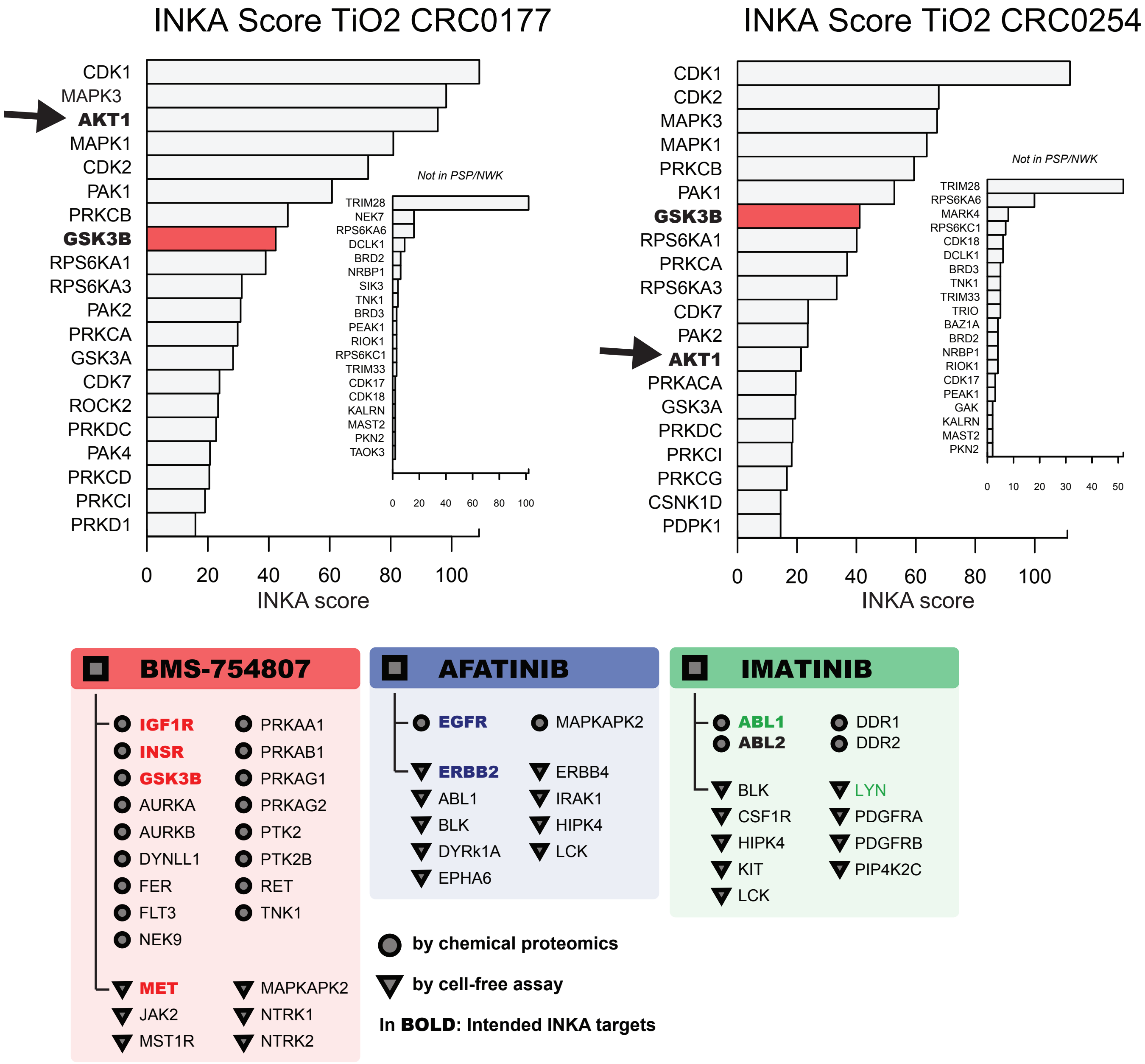

Appendix Figure S11C. INKA analysis of patient-derived xenograft (PDX) tissue subjected to pTyr-based and TiOx-based phosphoproteomics.

INKA score bar graphs generated using TiOx-based phosphoproteomic data for PDX models CRC0177 (upper left) and CRC0254 (upper right), as well as overview of targets of BMS-754807, afatinib, and imatinib with an affinity in the nanomolar range (lower). In the bar graphs with top 20 kinases, the relatively higher activity of AKT in CRC0177 relative to CRC0254 is indicated by an arrow and bold type. In the lower left panel, experimentally established targets in the nanomolar range are shown. Targets denoted by a circle were discovered using a chemical proteomics approach (Klaeger et al, 2017) and those denoted by a triangle were identified using cell-free assays (Carboni et al, 2009; Mulvihill et al, 2009; Davis et al, 2011b). Kinases in bold type are intended drug targets while kinases that are off-targets are given in plain type. Kinases with a phosphotyrosine-specific top 20 INKA score for models CRC0177 or CRC0254 are indicated by matching colors as in Fig 6A (coral: BMS-754807, blue: afatinib, green: imatinib). Of these, GSK3B is the only kinase that is also in the TiOx-specific INKA top 20 (see upper panel).

Data information: Data in upper panel are plotted for single-sample measurements.

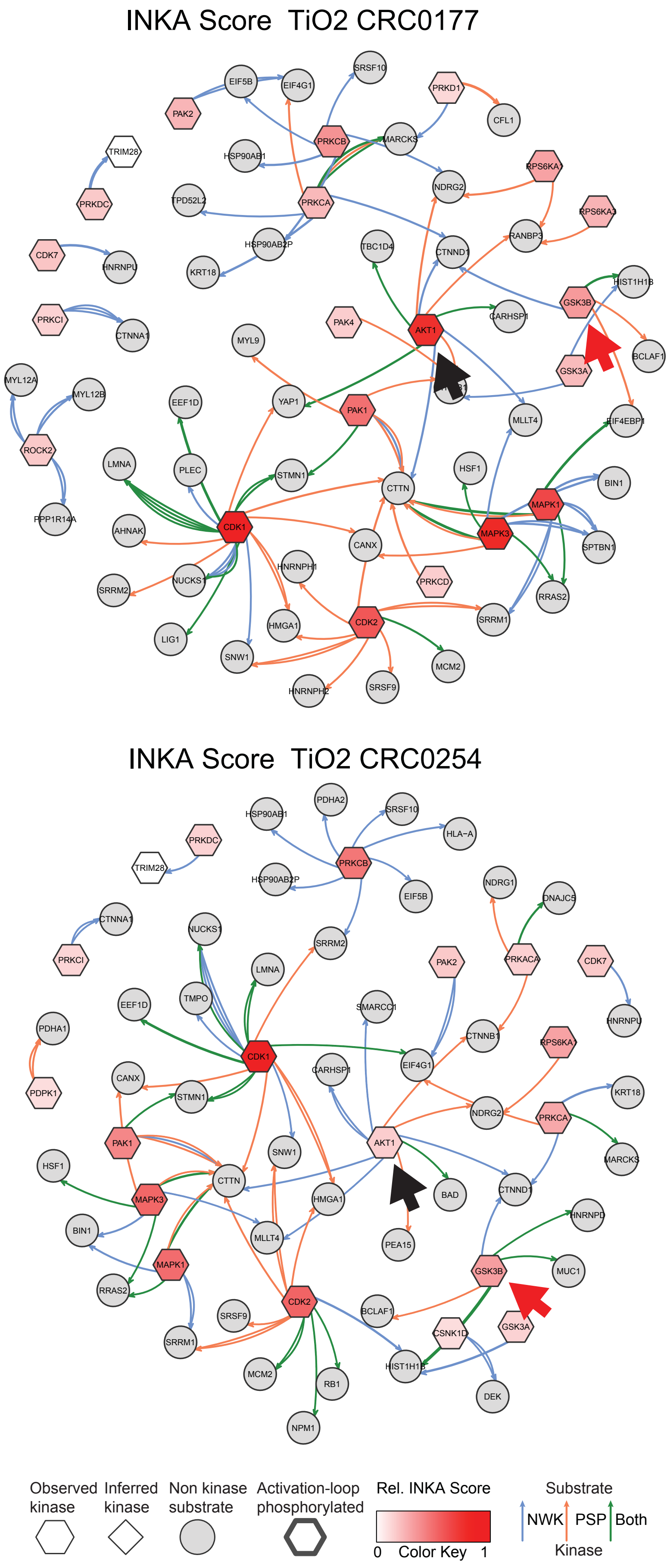

Appendix Figure S11D. INKA analysis of patient-derived xenograft (PDX) tissue subjected to pTyr-based and TiOx-based phosphoproteomics.

Kinase-substrate relation networks for the TiOx-based phosphoproteomic data on models CRC0177 (upper) and CRC0254 (lower). Large arrows indicate AKT1 (black), which is inferred to have higher activity in CRC0177 compared to CRC0254, and GSK3B (red), which is a target for BMS-754807 (red), respectively.

Data information: Data are plotted for single-sample measurements.

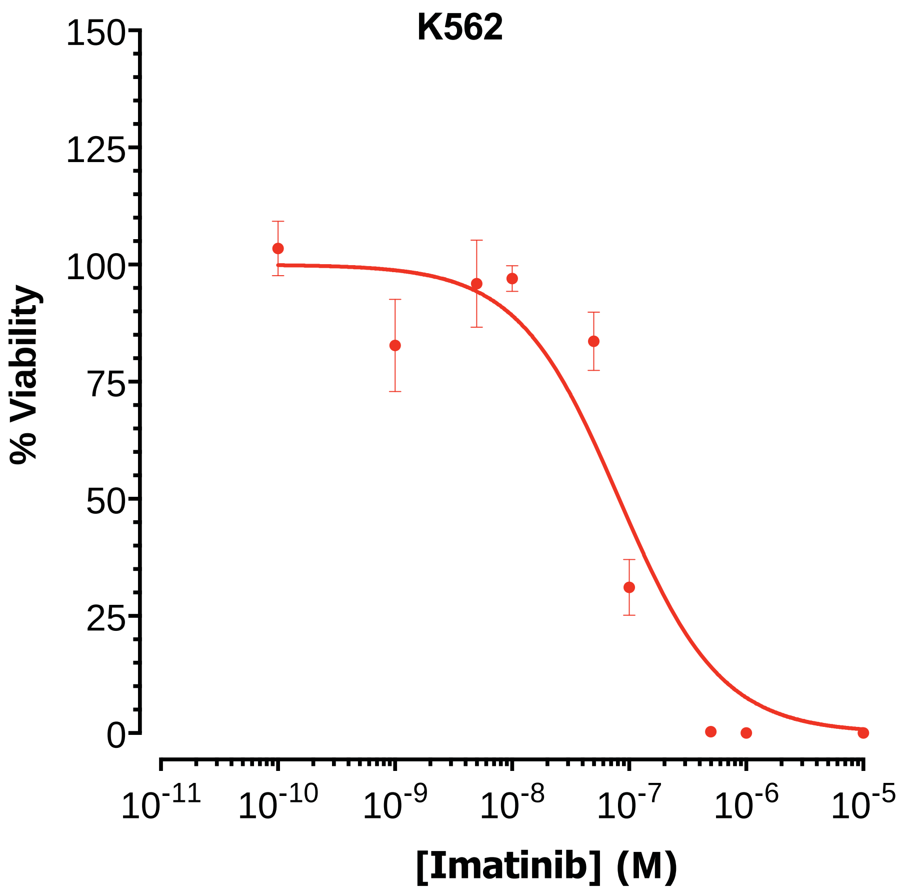

Appendix Figure S11E. Imatinib testing in positive control CML cell line K562.

K562 cells were cultured for 2-3 weeks, treated with imatinib for 7 days, and metabolic activity was measured by a luminescent ATP assay as a correlate of cell viability. Half-maximal inhibition occurred at IC50 = 82.3 nM with CI95 = 55 - 123 nM.

Data information: data are for 3 replicates.

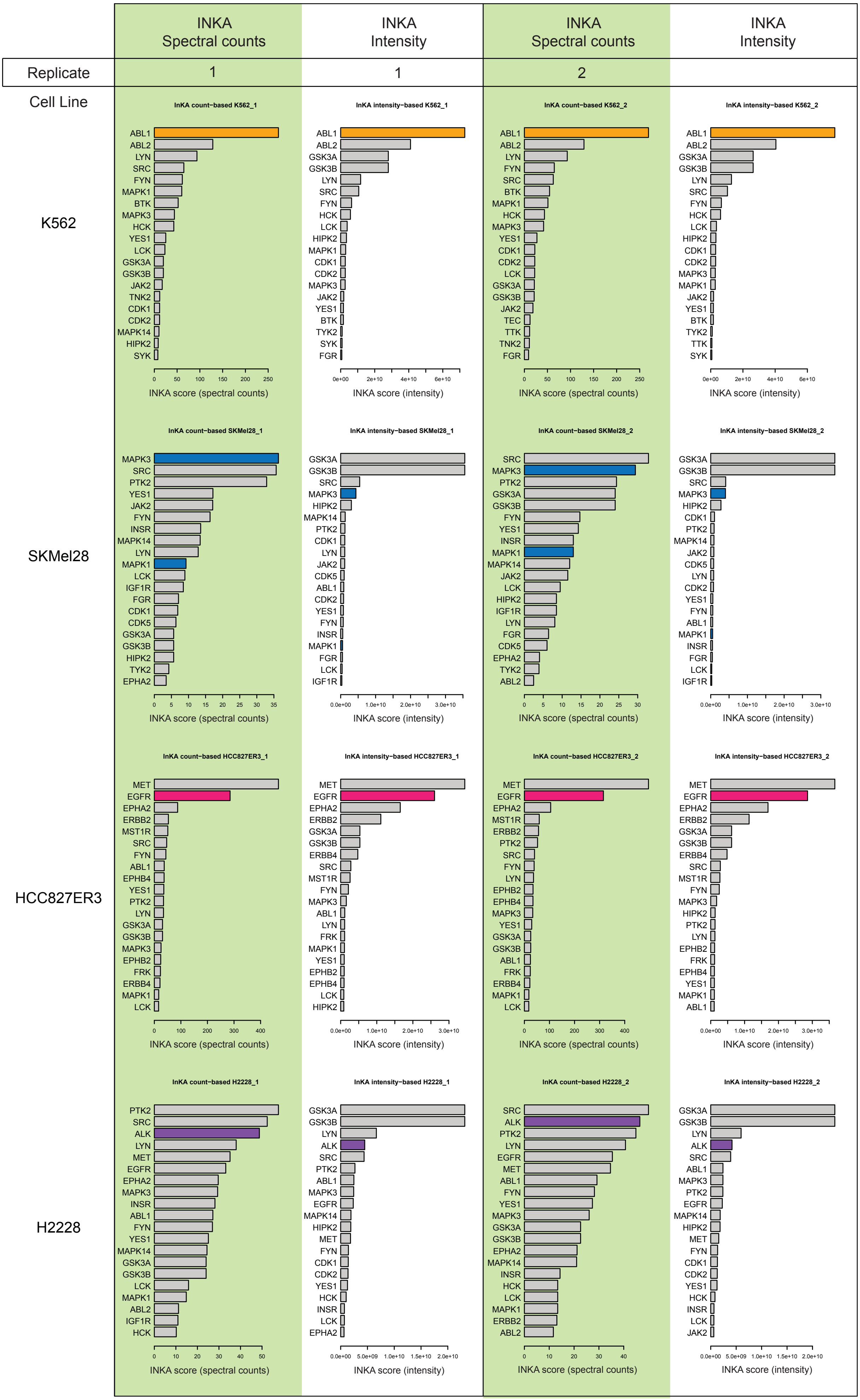

Appendix Figure S12. Comparison of count-based versus intensity-based INKA analysis of phosphoproteomics data on oncogene-driven cell lines.

Phosphotyrosine IP data for K562, SK-Mel-28, HCC827-ER3 and H2228 were analysed by INKA on the basis of spectral counts (green columns) and intensity (white columns).

Data information: there are duplicates for each cell line.

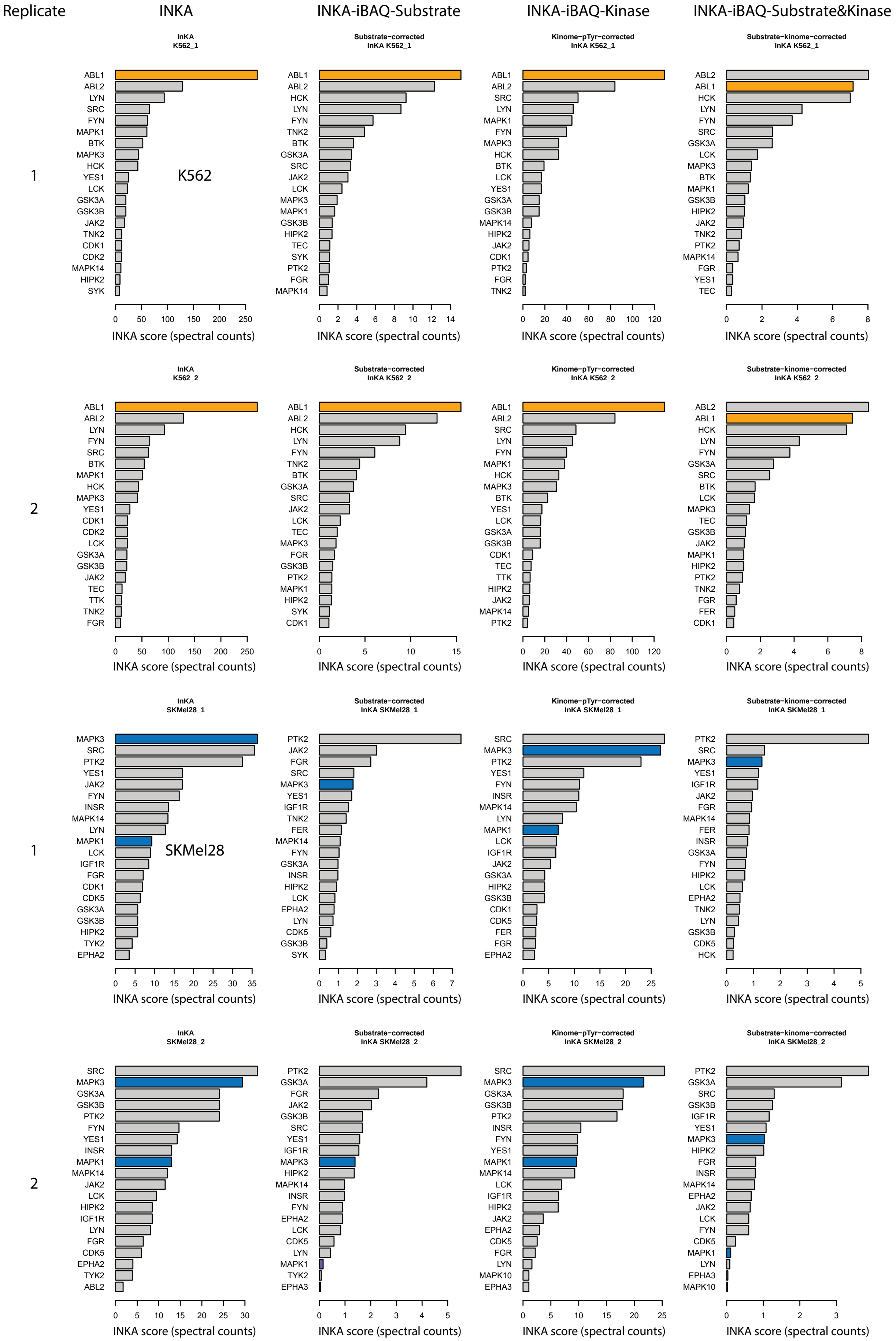

Appendix Figure S13. iBAQ-based INKA analysis of phosphoproteomics data on oncogene-driven cell lines. Part 1 of 3. Continues on next pages.

Phosphotyrosine IP data for K562, SK-Mel-28, HCC827-ER3 H2228, and U87 were analysed by INKA using IBAQ-corrected quantification of substrates, kinases and the combination of substrates and kinases using spectral counting data. **Shown here are data for K562 and SK-Mel-28.**

Data information: there are duplicates for each cell line (indicated by 1 and 2).

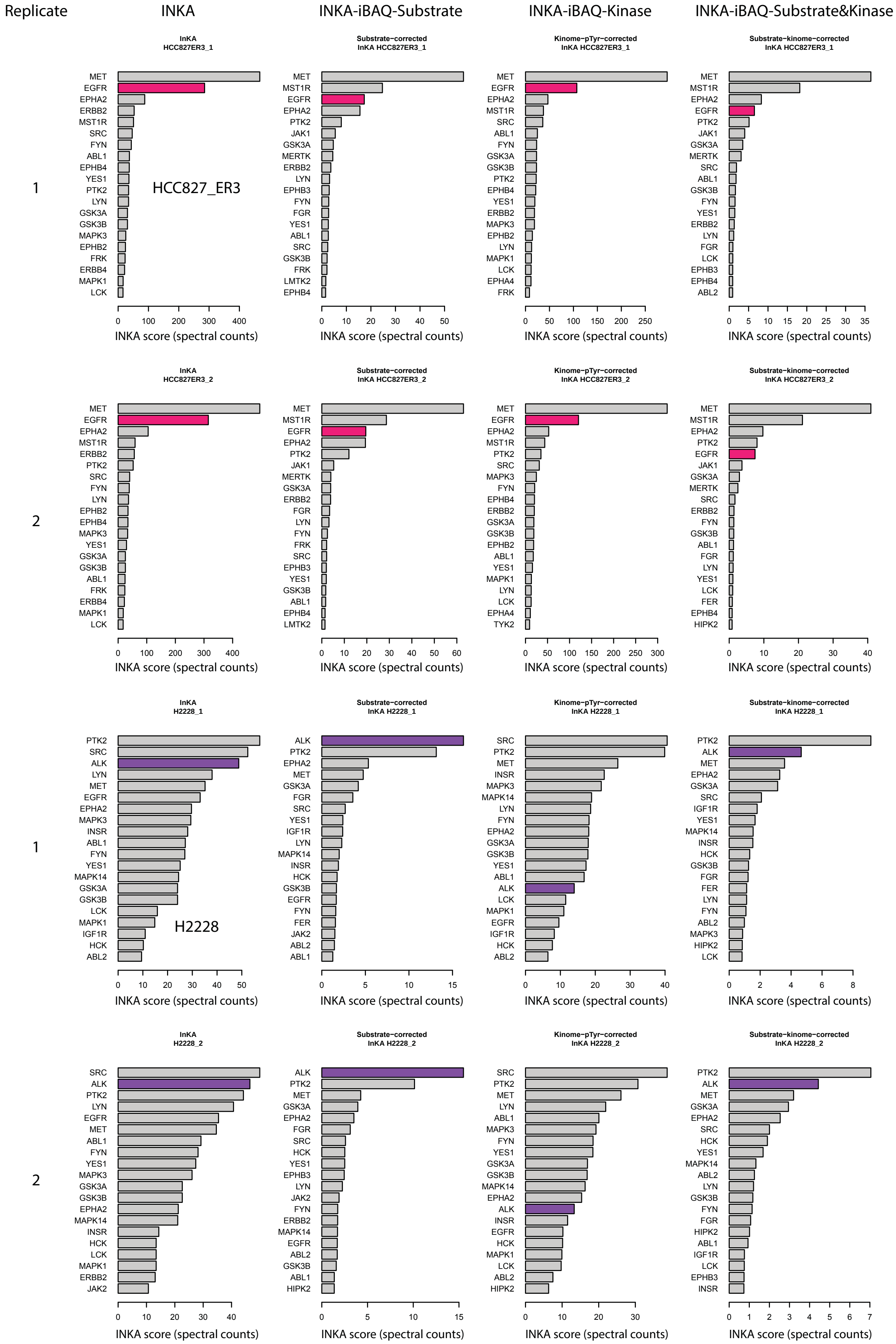

Appendix Figure S13. iBAQ-based INKA analysis of phosphoproteomics data on oncogene-driven cell lines. Part 2 of 3. Continues on next page.

Phosphotyrosine IP data for K562, SK-Mel-28, HCC827-ER3 H2228, and U87 were analysed by INKA using IBAQ-corrected quantification of substrates, kinases and the combination of substrates and kinases using spectral counting data. **Shown here are data for HCC827-ER3 and H2228.**

Data information: there are duplicates for each cell line (indicated by 1 and 2).

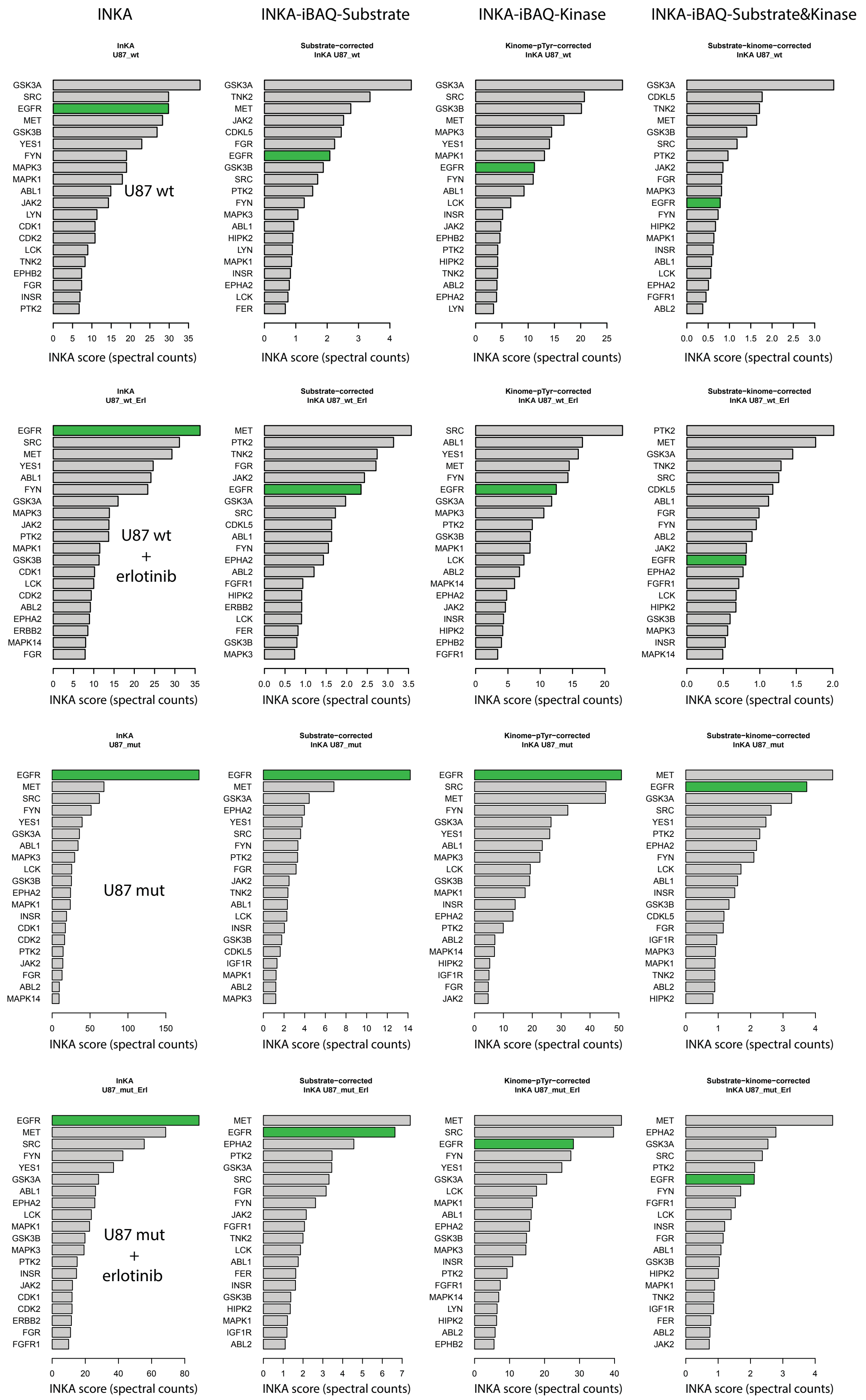

Appendix Figure S13. iBAQ-based INKA analysis of phosphoproteomics data on oncogene-driven cell lines. Part 3 of 3.

Phosphotyrosine IP data for K562, SK-Mel-28, HCC827-ER3 H2228, and U87 were analysed by INKA using IBAQ-corrected quantification of substrates, kinases and the combination of substrates and kinases using spectral counting data. **Shown here are data for U87 cells (wild type and mutant EGFRvIII) treated with erlotinib for 2 h.**

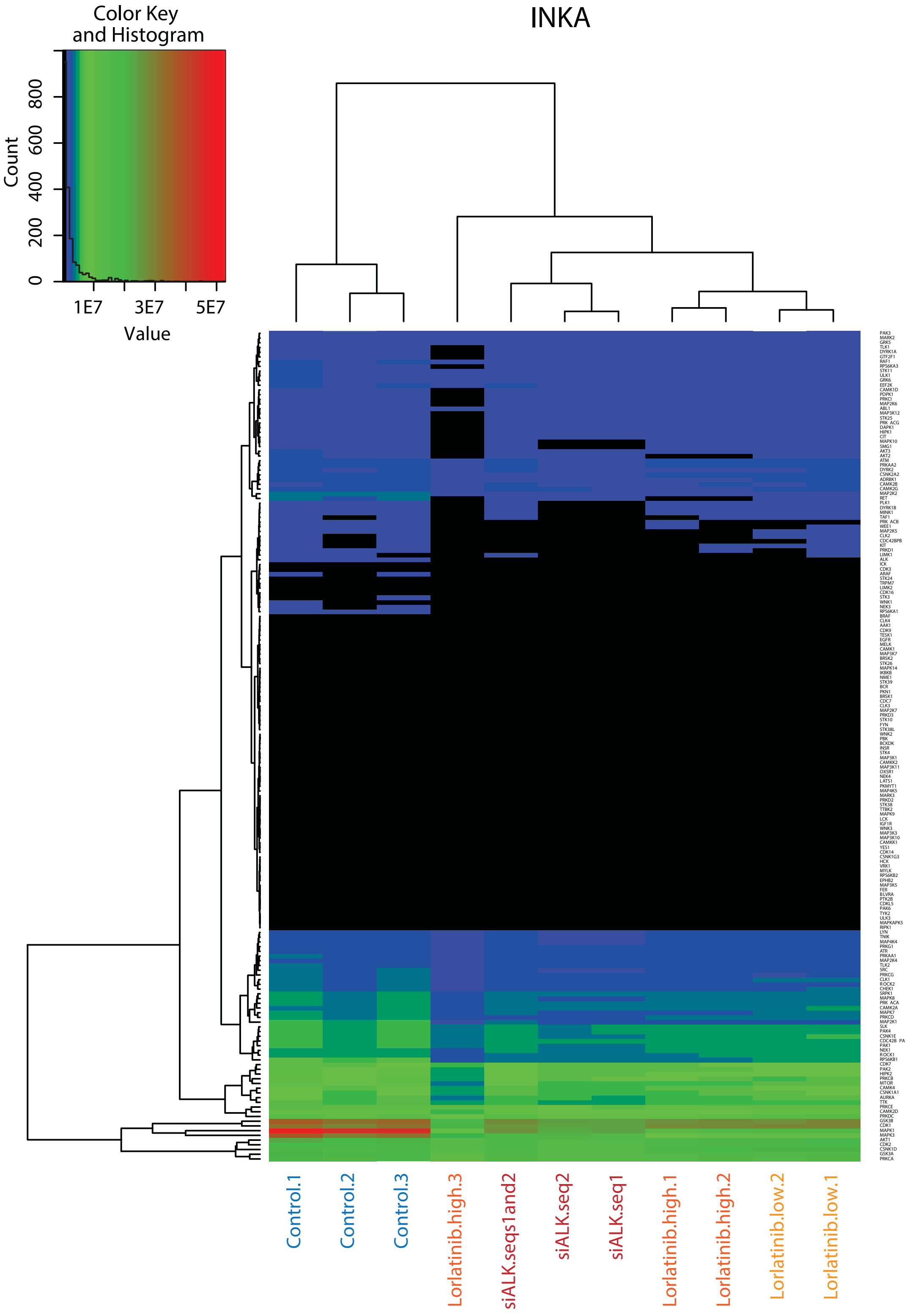

Appendix Figure S14A. INKA analysis of 11-plex TMT phosphoproteomics data of ALK signaling in neuroblastoma cells: unsupervised cluster analysis of INKA kinase activity ranking.

Data information: Analysis of dataset PXD009477 (Emdal *et al.*).

Control (*MYCN*-amplified and *ALK*-amplified) NB1 neuroblastoma cells versus siRNA *ALK* knockdown or loratinib-treated NB cells were analysed by phosphopeptide enrichment, 11-plex TMT labeling, and Orbitrap MS. Loratinib, an ALK inhibitor, was applied at high concentration (10 µM) or low concentration (10 nM).

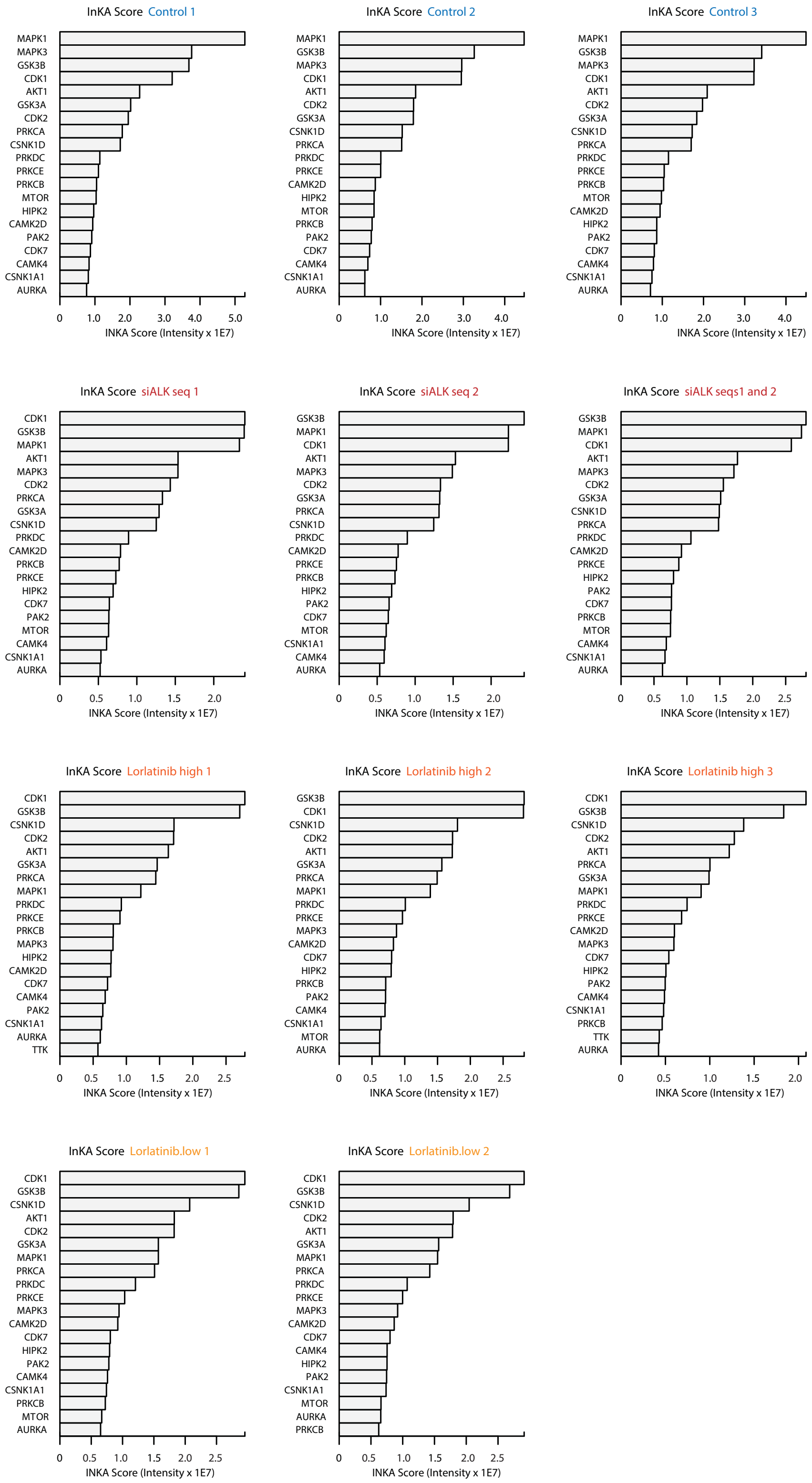

Appendix Figure S14B. INKA analysis of 11-plex TMT phosphoproteomics data of ALK signaling in neuroblastoma cells: sample-specific bar graphs of top 20 kinase INKA scores.

Data information: Analysis of dataset PXD009477 (Emdal *et al*.).
Control (*MYCN*-amplified and *ALK*-amplified) NB1 neuroblastoma cells versus siRNA *ALK* knockdown or loratinib-treated NB cells were analysed by phosphopeptide enrichment, 11-plex TMT labeling, and Orbitrap MS. Loratinib, an ALK inhibitor, was applied at high concentration (10 µM) or low concentration (10 nM).

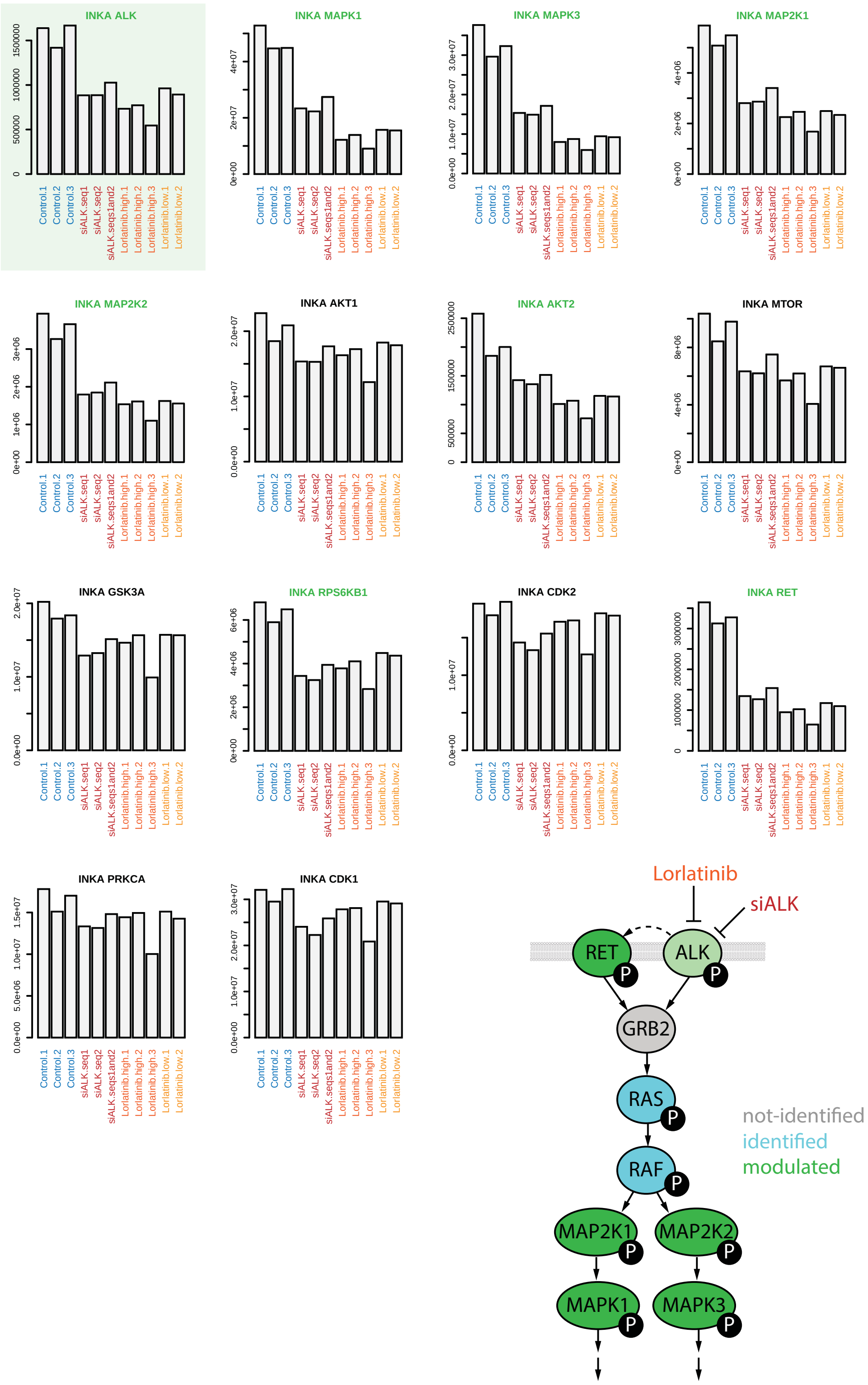

Appendix Figure S14C. INKA analysis of 11-plex TMT phosphoproteomics data of ALK signaling in neuroblastoma cells: kinase-specific bar graphs of INKA scores in individual samples and visualization of affected MAP kinase signaling network.

Data information: Analysis of dataset PXD009477 (Emdal *et al.*).
Control (*MYCN*-amplified and *ALK*-amplified) NB1 neuroblastoma cells versus siRNA *ALK* knockdown or loratinib-treated NB cells were analysed by phosphopeptide enrichment, 11-plex TMT labeling, and Orbitrap MS. Loratinib, an ALK inhibitor, was applied at high concentration (10 µM) or low concentration (10 nM).
